# Different susceptibility of mouse strains against Japanese encephalitis virus

**Published:** 2011-01-10

**Authors:** Kai Wang, Vincent Deubel

**Affiliations:** 1 Unit of Emerging Viruses, Institut Pasteur of Shanghai, Chinese Academy of Sciences, Shanghai Institute of Biological Sciences , Shanghai, 200025, PR China; 2 Institut Pasteur du Cambodge, Phnom Penh, Cambodia

## 

Japanese encephalitis is still the leading cause of viral encephalitis in Asia, where 30,000 to 50,000 cases are reported to WHO each year, resulting in an estimated 10,000 to 15,000 deaths annually. However, the majority of human infections with JEV are asymptomatic or very mild symptomatic and only about 1 in 250 infected persons develop clinical disease. Therefore, it is of importance to find out the cause of the different susceptibility with the comparison of different resistance towards JEV among various lab mice strain. We started with the observation of potential different organ tropism of JEV and distinct host immune response of JEV infection among resistant/susceptible (R/S) mice in vivo. Primary cells, including murine embryo fibroblast, bone marrow derived macrophage and bone marrow derived dendritic cells, were cultured in vitro to test specific host cell/virus interaction. We found that the virus levels in the bone marrow cells from R/S mice are significantly different.

